# Use of Favipiravir to Treat Lassa Virus Infection in Macaques

**DOI:** 10.3201/eid2409.180233

**Published:** 2018-09

**Authors:** Kyle Rosenke, Heinz Feldmann, Jonna B. Westover, Patrick William Hanley, Cynthia Martellaro, Friederike Feldmann, Greg Saturday, Jamie Lovaglio, Dana P. Scott, Yousuke Furuta, Takashi Komeno, Brian B. Gowen, David Safronetz

**Affiliations:** National Institutes of Health, Hamilton, Montana, USA (K. Rosenke, H. Feldmann, P.W. Hanley, C. Martellaro, F. Feldmann, G. Saturday, J. Lovaglio, D.P. Scott);; University of Manitoba, Winnipeg, Manitoba, Canada (H. Feldmann, D. Safronetz);; Utah State University, Logan, Utah, USA (J.B. Westover, B.B. Gowen);; Toyama Chemical Co., Ltd., Toyama, Japan (Y. Furuta, T. Komeno);; Public Health Agency of Canada, Winnipeg (D. Safronetz)

## Abstract

Lassa virus, the cause of Lassa fever in humans, is endemic to West Africa. Treatment of Lassa fever is primarily supportive, although ribavirin has shown limited efficacy if administered early during infection. We tested favipiravir in Lassa virus–viremic macaques and found that 300 mg/kg daily for 2 weeks successfully treated infection.

Lassa virus (LASV; family *Arenaviridae*, genus *Mammarenavirus*) is the etiologic agent of the severe hemorrhagic disease Lassa fever. Annually, ≈300,000 persons become infected with LASV, 20% of which experience life-threatening clinical manifestations including edema, hemorrhage, and multiorgan failure, resulting in an estimated 5,000 deaths (*1*). Most human infections are acquired from the natural rodent reservoir, the multimammate rat (*Mastomys natalensis*). Human-to-human transmission, mostly nosocomial, occurs (*1*). LASV has a relatively well-defined region of endemicity exclusive to West Africa. Incidence of LASV infections is highest in Nigeria, Sierra Leone, Liberia, and Guinea, although sporadic cases and moderate outbreaks of Lassa fever have been documented in many other West Africa nations (Technical Appendix Figure 1) (*2*). Over several decades, importation of Lassa fever into Europe, Asia, and the Americas has increased (*2*).

Treatment of Lassa fever is largely supportive, although ribavirin is used off label, despite side effects and limited efficacy data (*3*). Recently, the antiviral favipiravir (T-705; 6-fluoro-3-hydroxy-2-pyrazinecarboxamide) has gained attention as a broad-spectrum antiviral drug against RNA viruses (*4*) including LASV; initial studies using small animal models have been conducted (*5*,*6*). We assessed the antiviral efficacy of favipiravir in LASV-infected cynomolgus macaques; this animal model reliably recapitulates several hallmarks of Lassa fever infection in humans (*7*).

## The Study

We randomly divided 8 female cynomolgus macaques (*Macaca fascicularis*) into 2 groups of 4 each (treatment and control) and injected each animal intramuscularly with a lethal dose of LASV, strain Josiah (1 × 10^4^ 50% tissue culture infective dose [TCID_50_]) (Table). Treatment began at 4 days postinfection (dpi), 1 day after the onset of viremia (*7*). The initial treatment (300 mg/kg favipiravir) was administered intravenously; subsequent treatments (300 mg/kg favipiravir every 24 h for 13 d) were administered subcutaneously. The dosage was based on the successful treatment of Lassa fever in guinea pigs (*5*). To avoid the confounding effects of ribavirin, we did not give it in combination. LASV-infected control animals received an equivalent volume of vehicle by the same route and schedule. Animals were assessed twice daily; physical examinations, including hematologic, blood chemistry, and virologic assessments, were conducted regularly.

**Table:** Study design for treatment of Lassa virus infection in cynomolgus macaques*

Study, no. animals	Treatment	Frequency†	Dose, mg/kg	Loading dose, mg/kg	Total daily dose, mg/kg	Survived/total, no.
First						
4	Placebo	Every 24 h	Volume equivalent	Volume equivalent	Volume equivalent	0/4
4	Favipiravir	Every 24 h	300	300	300	4/4
Second						
4	Placebo	Every 8 h	Volume equivalent.	Volume equivalent	Volume equivalent	0/4
4	Favipiravir	Every 8 h	50	300	150	0/4

*All animals were challenged with a previously determined lethal dose of 10^4^ 50% tissue culture infective dose of Lassa virus (strain Josiah) via intramuscular injection. At day 4 after infection, a time coinciding with the earliest onset of detectable viremia, treatment with placebo or drug was initiated by intravenous injection of the loading dose followed by daily (days 5–17) subcutaneous dosing. Animals were examined daily for clinical signs of disease, and samples were taken for hematologic, blood chemistry, and virologic analyses at 12 times throughout the study, beginning on the day of virus challenge and ending on day 56 after infection. †For 14 d.

**Figure 1:**
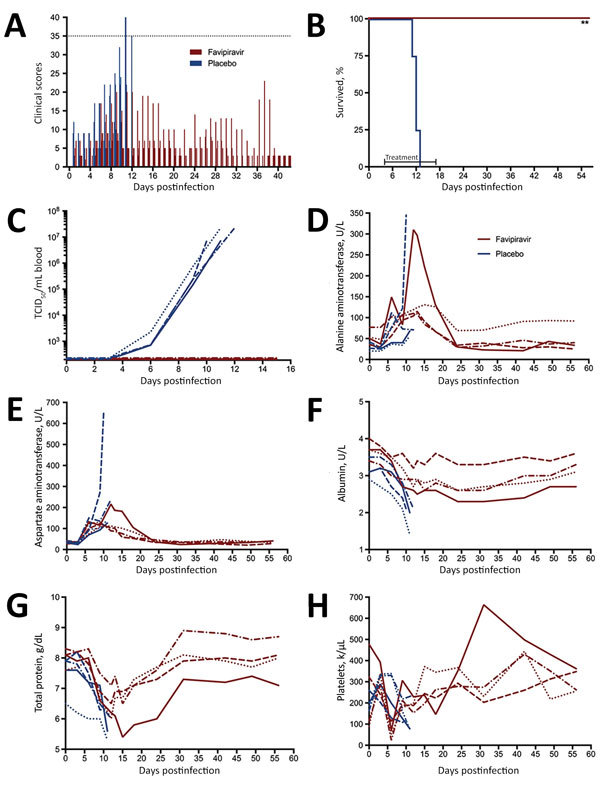
Effect of daily favipiravir treatments on morbidity and mortality rates, viral loads, and selected blood chemistry and hematology values during the course of the efficacy study in cynomolgus macaques challenged with 10^4^ TCID_50_ of Lassa virus. Groups of 4 animals each were given 300 mg/kg/d of favipiravir or placebo for 14 consecutive days, beginning on day 4 postinfection. A) Daily clinical scores (dotted line indicates euthanasia score of 35). B) Survival curve (*p<0.01 compared with placebo-treated animals by the Mantel-Cox log-rank test). C) Viremia as assessed by TCID_50_ assay. D) Alanine aminotransferase levels. E) Aspartate aminotransferase levels. F) Albumin levels. G) Total protein levels. H) Platelet levels. TCID_50_, 50% tissue culture infective dose.

Reduced activity and appetite, probably resulting from being anesthetized daily, was noted early in animals in both groups (Figure 1, panel A). At 6 dpi (2 d after treatment began), clinical scores for 3 of 4 animals in the treatment group plateaued and remained consistent for the remainder of the study. Although the score for the remaining animal was higher, all favipiravir-treated animals survived LASV challenge (p<0.01; Figure 1, panel B). In contrast, clinical scores for the control group increased dramatically after 8 dpi; all control animals displayed anorexia, hunched posture, piloerection, and lethargy (Figure 1, panel A). The control animals reached the humane endpoint and were euthanized on 10, 11, and 12 dpi (Figure 1, panel B).

Although we detected viral RNA by quantitative PCR as early as 3 dpi (online Technical Appendix Figure 2, panel A), at no time was infectious LASV isolated from blood (Figure 1, panel C) or postmortem tissue samples (data not shown) from animals in the favipiravir group. However, in the control animals, increased liver enzyme levels were detected at 6 dpi, coinciding with infectious LASV (Figure 1, panels D, E). One animal in the favipiravir group demonstrated moderately increased levels of alanine aminotransferase and aspartate aminotransferase at 6–12 dpi, which resolved after treatment cessation. In the control animals, albumin and total protein levels decreased dramatically throughout LASV infection; in the favipiravir-treated animals, these levels displayed a more moderate decrease before returning toward reference levels (Figure 1, panels F, G).

**Figure 2:**
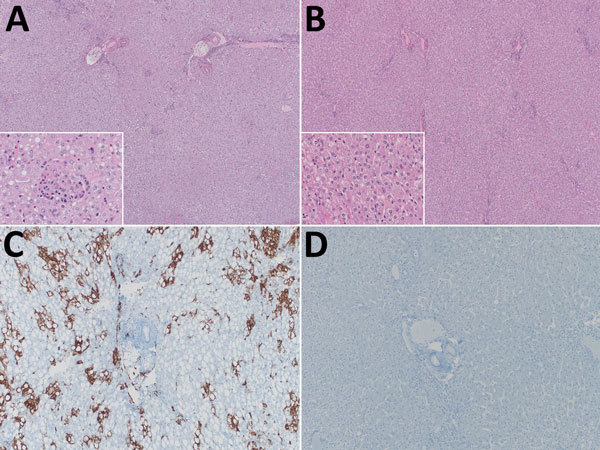
Histologic and in situ hybridization analyses of livers from cynomolgus macaques infected with Lassa virus (LASV) (1 × 10^4^ 50% tissue culture infective dose [TCID_50_]) and treated 1 time daily with favipiravir (treated) or vehicle only (control). A–B) Histologic analyses. A) Control, showing multifocal neutrophilic infiltrates with hepatocyte necrosis and degeneration (original magnification ×40). Inset shows hepatocyte necrosis (original magnification ×400). B) Treated, showing essentially healthy hepatic tissue (original magnification ×40). Inset shows healthy hepatic tissue (original magnification ×400). C–D) In situ hybridization analyses. C) Control, showing multifocal and coalescing viral RNA detected within hepatocytes (original magnification ×100). D) Treated, showing no viral RNA detected (original magnification ×100).

Hematologic profiles were less uniform between the 2 groups, although we observed several abnormalities previously associated with LASV infection (Technical Appendix Figure 3). After an initial increase, platelet counts in the control animals consistently decreased until euthanasia. In 2 of the favipiravir-treated animals, a similar increase was noted initially; however, a marked decrease occurred at 6 dpi (Figure 1, panel H). Platelet levels rebounded by 9 dpi and continued to increase. In addition, all 4 favipiravir-treated animals had lipemia (>301 mg/dL) during the treatment phase of the study (4–18 dpi).

Histopathologic examination of livers from control animals demonstrated hepatitis with neutrophil invasion and substantial steatosis, consistent with Lassa fever in this model (Figure 2, panel A) (*7*). In contrast, livers from favipiravir-treated animals showed no abnormalities (Figure 2, panel B). In situ hybridization detected LASV RNA within hepatocytes of control animals but not treated animals (Figure 2, panels C, D).

## Conclusions

Over the past 3 years, total numbers of Lassa fever cases and case-fatality rates among humans have increased (*8*). The current Lassa fever outbreak in Nigeria and Benin continues to show large numbers of Lassa fever cases and case-fatality rates >30% (*8*), again highlighting the lack of available treatments. Furthermore, the recent person-to-person transmission of LASV in Germany (*9*) serves as a reminder that Lassa fever is of global health concern.

Lassa fever patients are largely managed by supportive care in combination with ribavirin (*3*). Experimental efficacy of ribavirin was initially tested in a Lassa fever rhesus macaque model in which intramuscular injections 3 times daily for 14 days improved survival rates (*10*). In clinical trials in Sierra Leone, both oral and intravenous ribavirin increased survival rates of Lassa fever patients (*11*). Oral dosing of ribavirin has since remained the standard of treatment for Lassa fever, despite unproven efficacy from clinical studies (*3**,**12*). Moreover, side effects of ribavirin therapy have resulted in noncompliance (*12*).

The successful daily administration of favipiravir to macaques at an elevated dosing regimen of 300 mg/kg/d may partially explain the transient thrombocytopenia, elevated liver enzyme levels, and lipemia found in this study. Experimentally, favipiravir is a well-known broad-spectrum antiviral drug with in vitro inhibitory activity against a multitude of RNA viruses (*4*). In Japan, favipiravir is approved for influenza treatment; in the United States, phase 3 clinical trials for the same indication have been completed (*4*). During the West Africa Ebola outbreak, favipiravir was administered to humans on an emergency basis under a different dosing regimen, but effectiveness was limited (*13*). We therefore designed a second study that more closely followed the dosing in the Ebola trial (50 mg/kg every 8 h) (*13*). However, this multiple dose per day format failed to protect cynomolgus macaques from Lassa fever and did not alter disease progression (Table; Technical Appendix Figure 1, panel B, and Figure 4, panels A–D). Of note, the high-dose favipiravir therapy in macaques successfully abated the pathophysiologic parameters associated with LASV infection, resulting in survival. These results suggest that low doses of favipiravir have limited therapeutic effect, whereas higher doses are therapeutic and will improve clinical outcomes.

Favipiravir was recently administered in combination with ribavirin to successfully treat Lassa fever in 2 human patients. Although it is not possible to determine how effective favipiravir was in controlling these 2 cases, administration reduced viremia in both patients (*9*). Combination therapy with favipiravir and ribavirin in immunocompromised mice with LASV infection showed efficacy with suboptimal doses of each drug (*6*). The synergistic effect of the 2 compounds is also supported by several other studies in rodents (*14*). Recently, human monoclonal antibody therapy protected cynomolgus macaques from LASV infection (*15*). Combination therapy with favipiravir may be a future therapeutic strategy (*15*). On the basis of our findings, improved favipiravir tolerability in humans (*4*), availability of an oral formulation, and its advanced preclinical status (according to the US Food and Drug Administration), we recommend that favipiravir enter clinical trials as a treatment for Lassa fever.

Technical Appendix Additional information concerning study design and technical details pertinent to methods; supplemental data for results of the experiments described.
