# Supplementary material for: Use of Favipiravir to Treat Lassa Virus Infection in Macaques
Source: Emerg Infect Dis. 2018 Sep;24(9):1696–9. doi: 10.3201/eid2409.180233 (PMC6106425; doi:10.3201/eid2409.180233)
Supplement: Technical Appendix — Additional information concerning study design and technical details pertinent to methods; supplemental data for results of the experiments described. [file 18-0233-Techapp-s1.pdf]

Article DOI: https://doi.org/10.3201/eid2409.180233

Use of Favipiravir to Treat Lassa Virus Infection in Macaques

Technical Appendix

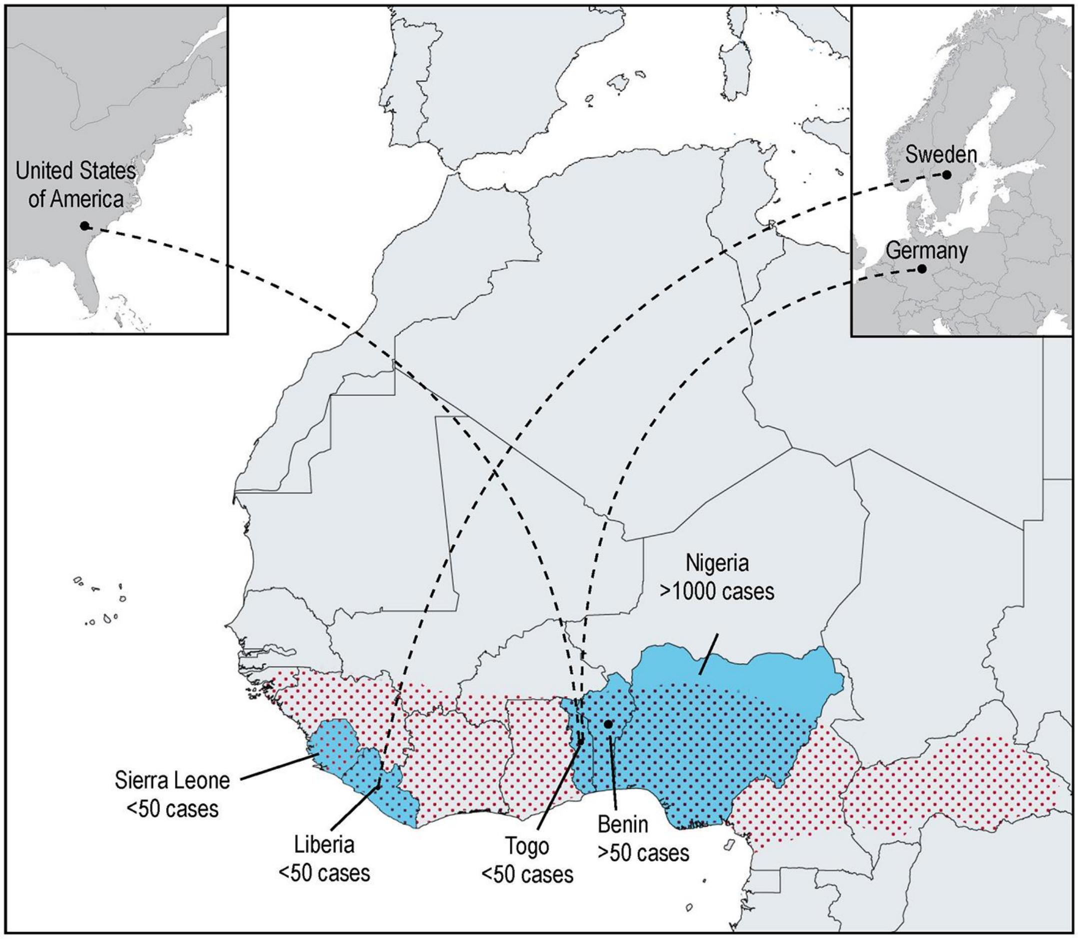

Technical Appendix Figure 1. Map illustrating reported cases of Lassa fever in West Africa since 2016. The countries in blue have all reported Lassa Fever cases since 2016. The red stipple identifies the Lassa virus endemic region. The dashed lines represent imported cases in 2016. The numbers indicate suspected cases, and suspected and confirmed deaths from each country (7-10).

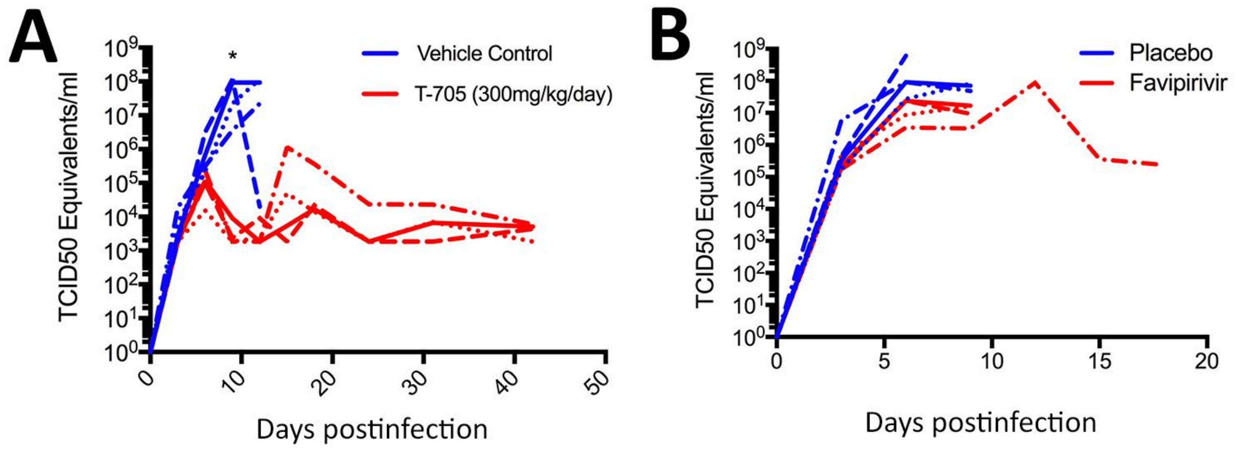

Technical Appendix Figure 2. Viral RNA (TCID50 equivalents) was detected by qPCR in all in animals in both experiments. (A) Viral RNA was detectable in both the treated and untreated animals in the successful 1x daily 300mg/kg/day study as early as 3 d.p.i.. However, there was a significant difference between the treated and untreated animals beginning at 8 d.p.i. ($P<0.05$) and infectious virus was never isolated from the favipiravir treated animals. (B) There was no significant difference in viral RNA detected by qPCR between the 3x daily 150mg/kg/day favipirivir treated animals and the placebo controls (*P* >0.05). P-values determined by Favipirivir treated animals compared to placebo-treated animals on days 9 and 12 by the Mann-Whitney nonparametric t test.

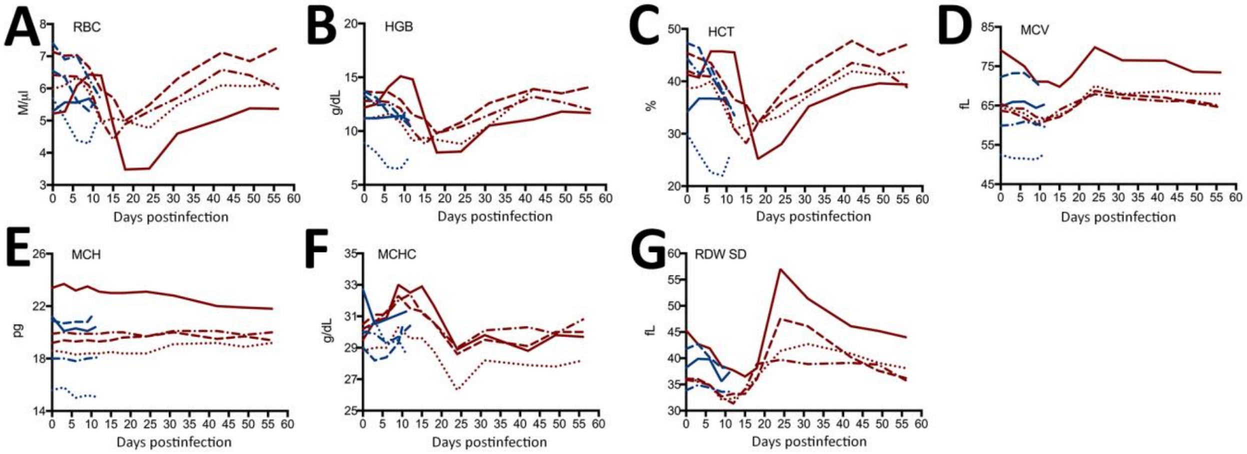

Technical Appendix Figure 3. Hematology values of the 1x daily favipiravir and placebo treated animals over the course of the experiment. Red blood cells (RBC), hemoglobin (HGB), hematocrit (HCT), mean corpuscular volume (MCV), mean corpuscular hemoglobin (MCH), mean corpuscular hemoglobin concentration (MCHC), red cell distribution width standard deviation (RDW-SD), and red cell distribution width coefficient of variation (RDW-CV).

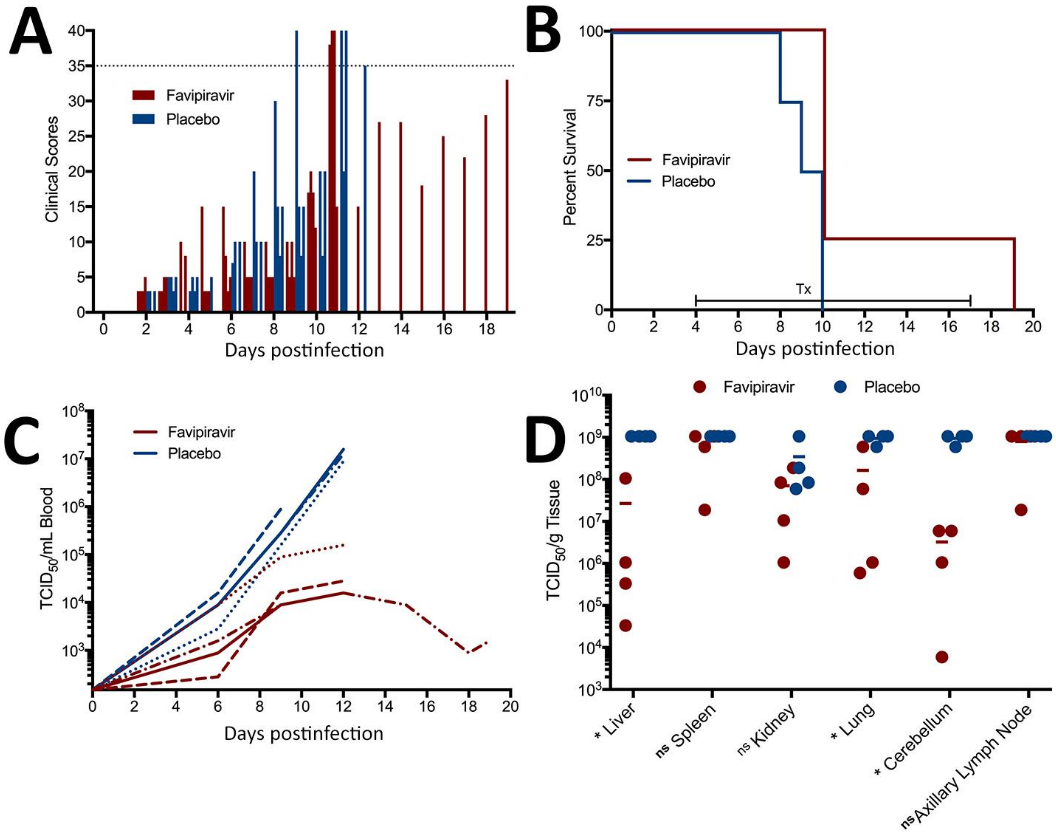

Technical Appendix Figure 4. Effect of 3x daily favipiravir treatment on morbidity, mortality and viral loads in cynomolgus macaques challenged with 10^4 $TCID_{50}$ LASV. Groups of animals (n=4) were treated (every 8 hours, 50mg/kg) for 14 consecutive days with 150mg/kg/day of favipiravir (red) or placebo (blue) beginning on day 4 post-infection. (A) Daily clinical scores (dotted line indicates euthanasia score of 35); (B) Survival curve, Tx = period of treatment; (C) Viremia and (D) Organ virus loads as assayed by $TCID_{50}$ assay. Infectious virus was readily detected in the blood with lower titers in the favipiravir-treated animals. Virus loads were high in several tissues with statistically significant lower titers in liver, lung and cerebellum of the favipiravir-treated animals. The symbol shapes represent the same animal across all tissues; *$P < 0.05$ compared to placebo-treated animals by the Mann-Whitney nonparametric t test; ns indicates tissues with no statistically significant difference.
